# Postmortem Presentation and Review of Danon Disease: A Rare Metabolic Disorder

**DOI:** 10.1155/crip/1525653

**Published:** 2026-10-12

**Authors:** Bryanna Rather Eguh, Antonio Farinhas, Shilpika Bagh, Rachael Connington, Stephanie Klinesmith

**Affiliations:** ^1^ Department of Basic Sciences Touro College of Osteopathic Medicine Touro University Middletown New York USA touro.edu; ^2^ Department of Biomedical Sciences College of Osteopathic Medicine Indiana University of Pennsylvania Indiana Pennsylvania USA iup.edu

**Keywords:** Danon disease, heart failure, mitochondrial disease

## Abstract

Danon disease is a rare, X‐linked dominant inherited lysosomal storage disorder that involves a mutation in the lysosomal‐associated membrane Protein 2 gene. Mutation in this gene results in severe disease clinical manifestations that may include skeletal and cardiac myopathies, retinopathies, and intellectual disabilities. This case report explores postmortem findings observed in a 25‐year‐old male donor who succumbed to heart failure secondary to Danon disease. With no transplant history, postmortem findings include hypertrophy, extensive fibrosis, and vacuolization in cardiomyocytes compared with expected anatomical findings in an unaffected patient. This case report is aimed at deepening the understanding of the clinical manifestations of Danon disease, raise awareness, and explore diagnostic approaches and differentials to help identify additional treatment options that may improve patient outcomes.

## 1. Introduction

Danon disease (DD) is a rare lysosomal storage disorder involving a mutation of the lysosome‐associated membrane protein 2 (LAMP‐2) gene encoded on the X chromosome [1]. As such, it is most common and most severe in young males and can manifest as retinopathies, intellectual disabilities, and cardiac and skeletal muscle abnormalities. In males, the most common clinical finding is the development of concentric left ventricular hypertrophy (LVH) in childhood, which ultimately requires a heart transplant. Females, however, typically develop dilated cardiomyopathy later in disease progression than males [2]. Males with DD typically show onset of symptoms by 12 years of age, with advanced disease likely leading to cardiac transplantation or death by 19 years of age [2]. This article describes a rare case of a 25‐year‐old male with DD who exhibited postmortem findings consistent with LVH.

## 2. Case Presentation

This case report presents a 25‐year‐old male with no history of transplantation who succumbed to heart failure secondary to DD, per available body donation documentation. The donor was dissected as part of the routine gross anatomy curriculum at Touro College of Osteopathic Medicine in Middletown, New York, during the 2024–2025 academic year. The donor′s heart was removed following the dissection instructions outlined in *Grant′s Dissector*. The dissected heart was weighed and measured, revealing evidence of significant hypertrophy. The heart weighed 513 g with a circumference of 29.2 cm at the coronary sulcus, length of 16.3 cm from the aorta to the apex, and a left ventricular wall thickness of 2.1 cm. Other gross findings include a mildly pigmented green liver and densities in the kidneys.

Cross‐sectional analysis of the donor′s heart revealed biventricular hypertrophy and myocardial fibrosis. Figure 1a,b illustrate the markedly narrowed ventricular lumens compared with a normal heart, as demonstrated in a standard dissection from *Gray′s Anatomy*. Tissue samples were collected from the donor′s heart and stained with hematoxylin and eosin (H&E) and trichrome stains; see Figures 1, 2, 3, 4, 5 and 6.

**Figure 1:**
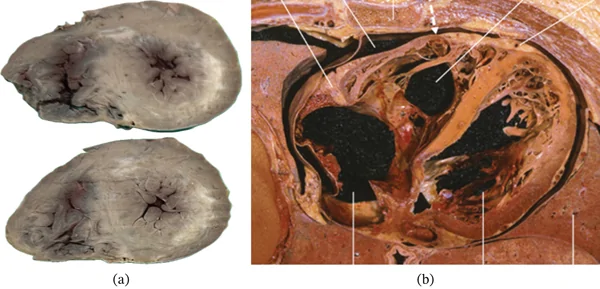
(a) Cross sections of explanted heart showing biventricular hypertrophy and fibrosis. (b) An axial section of the heart. *Note:* From *Gray′s Anatomy: The Anatomical Basis of Clinical Practice* (42nd ed., Figure 57.6c, p. 1157), by H. Gray, W. H. Lewis, and J. W. Rohen (Eds.), 2020, Elsevier.

**Figure 2:**
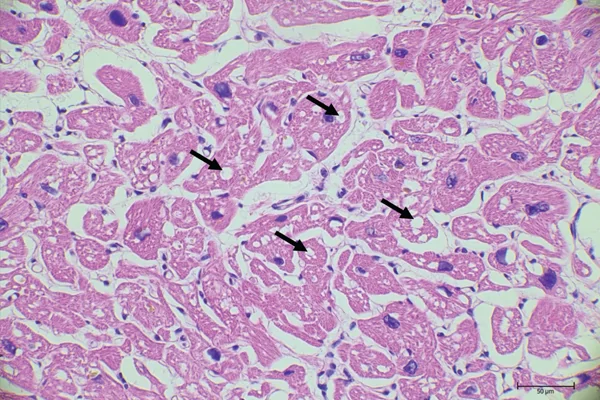
Photomicrograph (H&E) showing extensive vacuolization (arrows) of cardiomyocytes.

**Figure 3:**
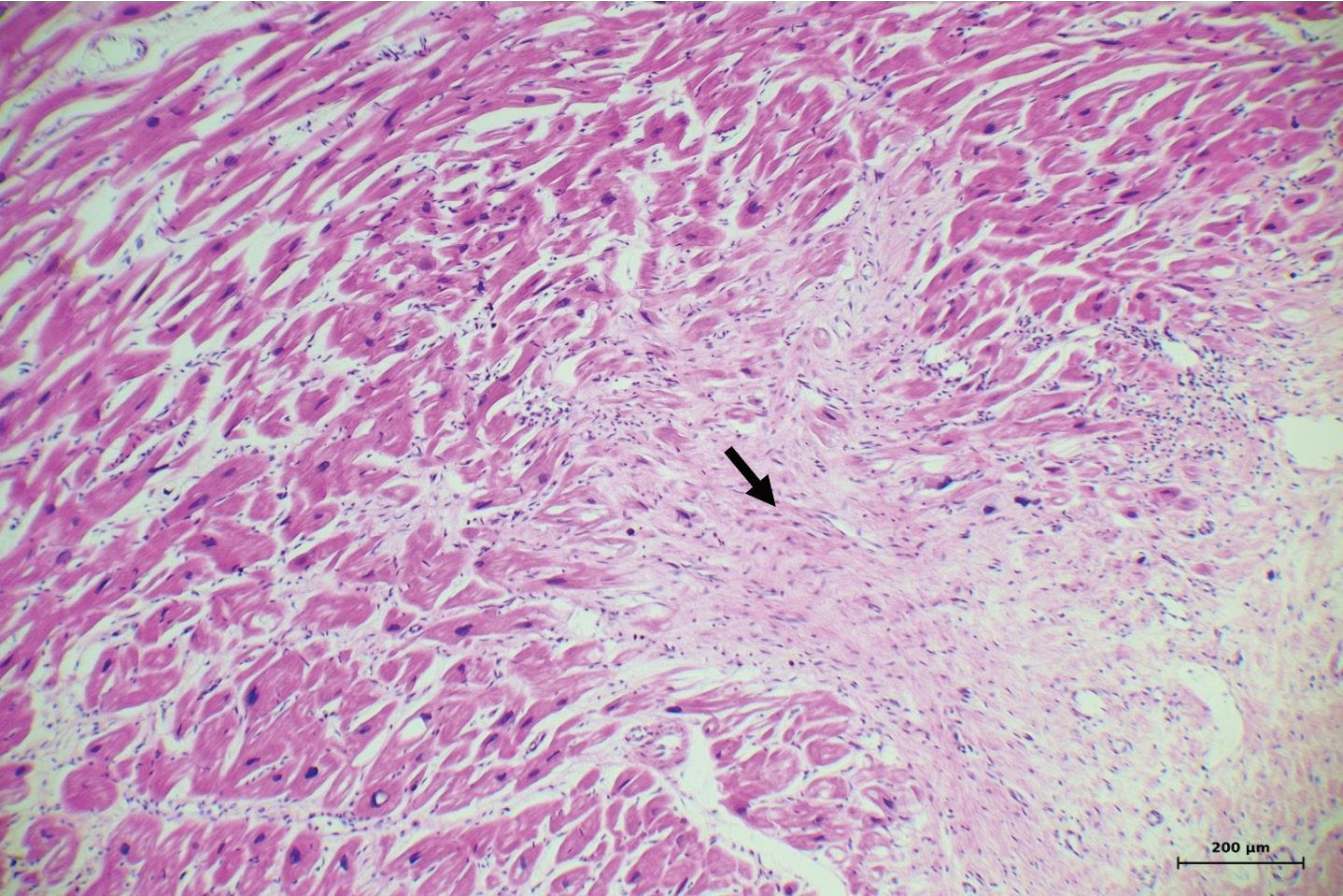
Photomicrograph (H&E) showing extensive fibrosis (arrow).

**Figure 4:**
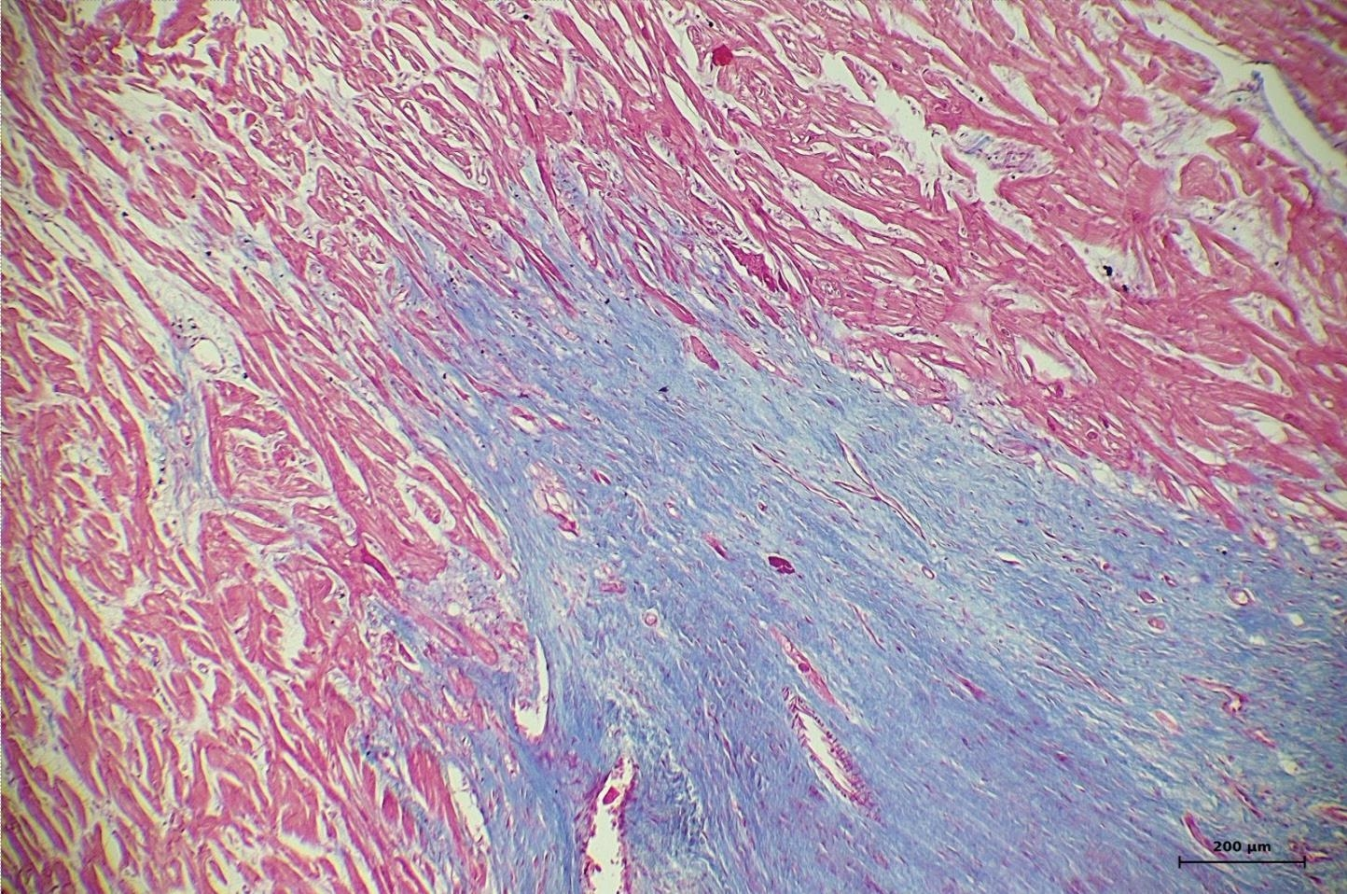
Photomicrograph (trichrome) highlighting marked fibrosis.

**Figure 5:**
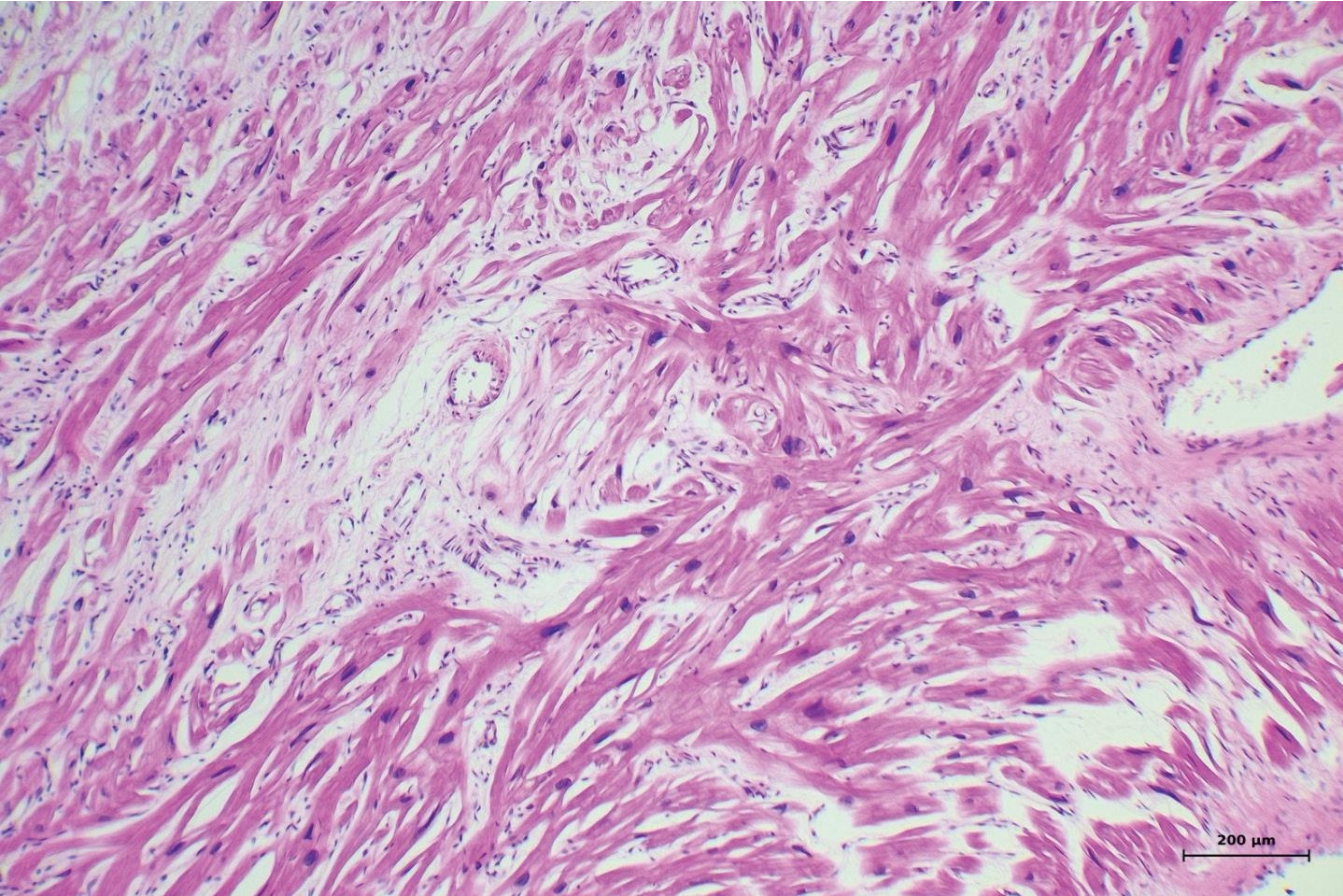
Photomicrograph (H&E) showing myocyte hypertrophy and disarray.

**Figure 6:**
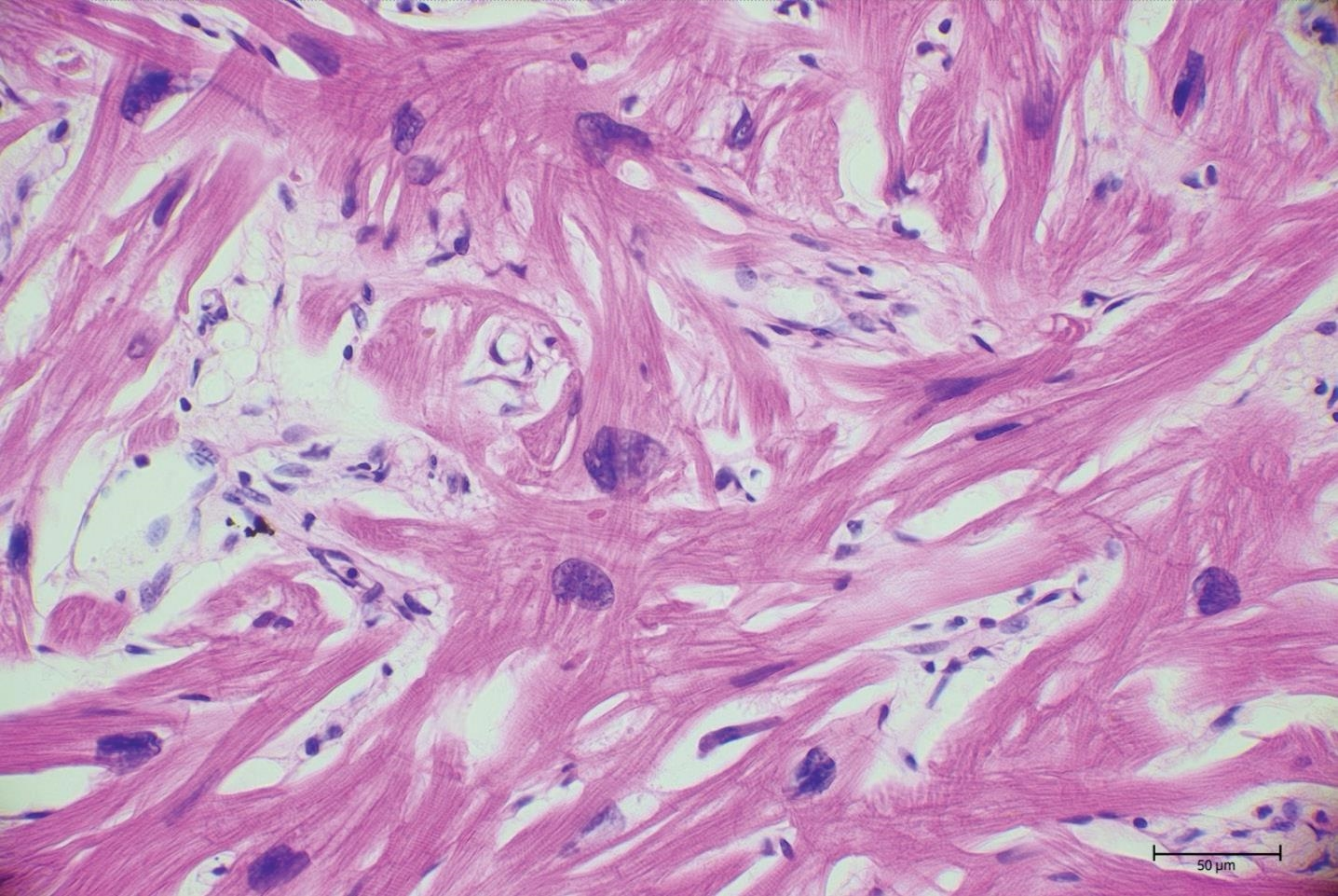
Photomicrograph (H&E) showing myocyte hypertrophy and disarray.

## 3. Discussion

DD is diagnosed based on clinical symptoms, genetic testing, and muscle biopsy. Male patients are often diagnosed in early childhood; family history of DD diagnosis is noted in 48% of cases, largely because of the disease′s X‐linked dominant inheritance pattern [3]. Although onset and progression are delayed in females, clinical presentation with cardiac conduction abnormalities is evaluated and treated similarly for males and females; diagnostic testing includes genetic testing and muscle biopsy to evaluate for gapping and glycogen presence. Previous studies have shown that affected males are unlikely to survive beyond the age of 25 without cardiac transplantation, with a literature review indicating an average life expectancy of 19 years [2].

### 3.1. Genetic Component

DD is a rare X‐linked dominant inherited genetic disorder that primarily affects multiple systems, including cardiac, pancreatic, hepatic, muscular, and nervous systems [1]. DD is caused by mutations in the LAMP‐2 gene, which encodes a protein involved in the recycling of cellular waste products within lysosomes. Most recent studies, such as one conducted by Gurka et al. [4], estimate that the DD population prevalence is less than 1 in 1,000,000 and that fewer than 1,000 cases are diagnosed annually worldwide. The results of this study revealed that the rate of DD is significantly higher in specific at‐risk groups. Specifically, Gurka et al. [4] identified that individuals with LVH, especially those with specific cardiac features like pre‐excitation, were found to have DD. Early recognition in higher risk patients is critical for genetic counseling and management planning.

The LAMP‐2 protein is a family of membrane glycoproteins that provide selectins and carbohydrate ligands and is located on chromosome Xq24 [5]. The function of LAMP‐2 proteins includes protection, maintenance, and adhesion of lysosome proteins and properties that break down and recycle cellular materials. Specifically, these proteins aid in autophagic vacuole fusion with lysosomes, endocytic receptors on dendritic cells, reactive oxygen clearance, and cell death. Alternative splicing of this gene results in multiple transcript variants encoding distinct proteins that are all related to lysosomal properties within different tissues [5]. The mutated LAMP‐2 seen in DD correlated with blockage of autophagic activity by impeding fusion events of autophagosome–lysosomes, which results in inefficient lysosome biogenesis and maturation [1]. This leads to an inability to degrade and reuse engulfed cellular substances. As a result, glycogen granules and intracytoplasmic vacuoles containing autophagic material accumulate, causing musculoskeletal impairments and improper cellular functions that may be observed as micro and macroscopic pathologies.

As DD follows an X‐linked dominant inheritance pattern, genetic males typically have only one X chromosome and are therefore at an increased likelihood of developing the disease compared with females, who typically have two X chromosomes [5, 6]. Thus, a mutation in a male′s single LAMP‐2 gene typically leads to severe, early‐onset disease with rapid progression, especially involving cardiomyopathy and muscle weakness [2]. In contrast, typical genetic females have two X chromosomes, so the unaffected X chromosome can partially compensate for the mutated LAMP‐2 gene [7]. Clinical manifestations in females can be explained by X‐inactivation, or lyonization, where one of the two X chromosomes in each cell is randomly inactivated. As a result, in some cells, the healthy copy of the LAMP‐2 gene is active, and in others, the mutated one is. This mosaic expression results in less severe symptoms and slower progression of the disease in females compared with males [1, 7].

### 3.2. Clinical Signs and Symptoms

Management of DD varies depending on the manifestation of symptoms. In DD, the progression of cardiac manifestations is the greatest cause of mortality. Symptoms of DD often present in males earlier with more extracardiac manifestations than in women [1]. Additionally, males are more likely to exhibit concentric LVH with a normal ejection fraction, whereas females are more likely to exhibit dilated LVH [1, 7]. The exact causes for such differences in clinical manifestations are still being explored. Several hypotheses suggest that these sex‐specific differences may be influenced by estrogen, which enhances autophagy and lysosomal pathway activity in multiple cell types, thereby potentially providing a relative protective effect in females [8].

Additional organ systems may be affected, including hepatosplenomegaly, skeletal myopathies, and retinopathies that include pigmentary atrophy, bull′s‐eye maculopathy, color vision deficiencies, loss of visual acuity, blurred vision, and eye floaters or flashes. Other ocular findings include retinal degeneration, fine lens opacities, bilateral peripheral band of chorioretinal atrophy, cystoid macular edema, and foveal retinal pigment epithelial clumping [9].

In this case, there was evidence that the body donor exhibited severe cardiomyopathy and ocular dysfunction. The average adult male heart weighs 300 g, has an average circumference of 9 cm at the sulcus, an average length of 12 cm, and an average left ventricular wall thickness of 8.7 mm at the basal septum [6]. The findings in this report demonstrate that, compared with the average male, this male body donor had a heart that was 71% heavier, 224.4% larger in circumference, 35.8% longer aorta to apex, and had a left ventricle that was 2313.7% thicker. Because of defective autophagy, which is pathognomonic of DD, myocardial injury and fibrosis develop over time, as evident by chronically elevated troponin levels. Arrhythmias subsequently arise because of cardiomyocyte damage. In nearly 60% of individuals, supraventricular tachycardias (i.e. atrial flutter and fibrillation) and ventricular arrhythmias occur and ultimately lead to heart failure [10]. Unfortunately, cardiac arrest may be the first disease presentation in both males and females.

### 3.3. Treatment and Patient Outcomes

Currently, there is no cure for DD. However, multiple treatment modalities can help manage symptoms and improve the quality of life for affected individuals. Management guidelines for cardiomyopathy secondary to DD include echocardiography every 1–2 years with more frequent imaging as symptoms progress, an annual electrocardiogram, measurement of serum B‐type natriuretic peptide (BNP), and Holter monitoring if arrhythmias develop [2]. Cardiac magnetic resonance (CMR) imaging is a useful tool to evaluate hypertrophy and track the progression of fibrosis. Treatment approaches for heart failure secondary to cardiomyopathy include fluid volume management and an implantable cardioverter‐defibrillator (ICD) if arrhythmias develop. Medications to treat cardiac conduction abnormalities and improve heart function, such as guideline‐directed medical therapy for heart failure, may also be useful in patients who are symptomatic [2, 11].

Ultimately, the definitive treatment for LVH and heart failure secondary to DD is prompt cardiac transplantation once symptoms progress, left ventricular ejection fraction (LVEF) decreases, or CMR imaging demonstrates fibrosis greater than 30 mm [2, 12]. A decrease in LVEF is life‐threatening because it indicates that the heart is unable to pump enough blood to meet the body′s demands, ultimately resulting in poor cardiac output and poor end‐organ perfusion. The body compensates for the lack of perfusion by activating the renin–angiotensin–aldosterone system, which promotes vasoconstriction and sodium and water retention to maintain blood pressure, increase preload, and increase afterload. Over time, the increased afterload antagonizes the heart as it increases the resistance that the heart is pumping against. Further, the prolonged increase in preload can cause pulmonary congestion, resulting in dyspnea and lower extremity edema. Eventually, LVEF drops critically low, and the heart fails to generate enough pressure to maintain circulation, leading to hypotension, end‐organ failure, cardiogenic shock, and death.

Despite not curing the disease, one study demonstrated that in a cohort of patients with DD, the posttransplant 5‐year survival rate was 87.1%, demonstrating that transplant offers promising improvements in cardiac function and overall survival. Challenges patients face posttransplant include typical immunosuppression‐related complications such as opportunistic infections or transplant rejection. However, Hong et al. [12] detailed rapidly progressive muscle weakness in a DD patient 1 week after a heart transplant. The patient′s immunotherapy course consisted of tacrolimus and methylprednisolone—the latter having been implicated in accelerating myopathy in patients with DD. As such, methylprednisolone was discontinued, which resulted in the resolution of elevated creatinine kinase values [13, 14]. Therefore, it is imperative to factor in the adverse effects that posttransplant DD patients face, not only those associated with an underlying immunosuppressed state but also adverse events associated with DD itself.

Current research includes a Phase II clinical trial that is currently underway to evaluate the efficacy of RP‐A501, a recombinant adeno‐associated virus Serotype 9 (AAV9) vector carrying the LAMP‐2B transgene, in slowing cardiac disease progression in DD patients. In the preceding Phase I study, seven DD patients received a single infusion of RP‐A501 along with a transient immunosuppressive regimen consisting of prednisone, tacrolimus or sirolimus, and rituximab. Over a follow‐up period of 24 to 54 months, six patients demonstrated cardiac LAMP‐2 protein expression and showed evidence of either reduction or stabilization in left ventricular mass index, preserved LVEF, and reduced or stabilized levels of cardiac Troponin I and BNP [14]. This highlights the potential role of immunotherapies and other nonoperative approaches in slowing the progression of cardiac involvement in DD patients.

### 3.4. Limitations

This report is not without limitations. Because of the body donation company′s policies, additional clinical details, such as age of diagnosis, documented clinical findings, ongoing treatment, and family history, were unavailable. Only age and cause of death, per the death certificate, were provided. Next, the condition of the preserved tissue precluded optimal periodic acid–Schiff (PAS) staining. Because the donor tissue was obtained from a tertiary source, logistical constraints limited the time available for tissue processing before the donor had to be returned. In addition, postmortem formalin fixation likely reduced glycogen preservation, further compromising PAS staining quality. Despite these limitations, vesicular glycogen stores were still identified on Masson′s trichrome staining, providing supportive histopathologic evidence of glycogen accumulation. This difference in staining quality between the two tests is likely due to differences in staining technique and reagents. Finally, this case report is based on a single patient specimen. Consequently, these observations cannot be generalized to the broader population of patients with DD.

## 4. Conclusion

DD prognosis is contingent upon symptom severity and management. Affected males are believed to have a shorter life expectancy of 19 years of age because of glycogen accumulations found in cardiac myocytes that ultimately cause heart failure [2]. Affected females are reported to have a longer life expectancy of 34 years [2]. The goal of this report is to further understand clinical manifestations of DD, heighten awareness, investigate diagnoses, and provide differential possibilities so that additional treatments may be explored to improve patient outcomes. Secondary factors that may continue to be explored in order to explain the sex discrepancy of DD patient outcomes include investigating the protective roles of estrogen on cardiovascular and lysosomal function, as well as sex differences in mitochondrial function that could interact with the lysosomal defects in DD. Both of these topics remain areas of active research that are not yet fully understood.

## Funding

No funding was received for this manuscript.

## Ethics Statement

The subject of this case report was donated to Experience Anatomy and released to Touro College of Osteopathic Medicine for anatomical study and educational research in accordance with the Revised Uniform Anatomical Gift Act, G.S. § 130A‐412.3 et seq. and as authorized by North Carolina state law.

## Conflicts of Interest

The authors declare no conflicts of interest.

## Supporting information


**Supporting Information** Additional supporting information can be found online in the Supporting Information section. File S1: CARE checklist of information to include when writing a case report.

## Data Availability

The data that support the findings of this study are available on request from the corresponding author. The data are not publicly available due to privacy or ethical restrictions.
